# Pathways to effective network governance: A fuzzy-set QCA study of tripartite collaboration efficiency with Chinese official Weibo data

**DOI:** 10.1371/journal.pone.0331007

**Published:** 2025-10-07

**Authors:** Yuanzhuo Wu, Yifen Xia

**Affiliations:** 1 School of Public Policy and Management, Nanchang University, Nanchang, China; 2 Jiangxi Provincial Institute of Regional Economy, Nanchang University, Nanchang, China; BRAC Business School, BRAC University, BANGLADESH

## Abstract

In response to the insufficient research on multiagent collaboration mechanisms in existing network governance studies, this paper identifies the key influencing factors of the tripartite collaboration efficiency using the Grey Relational Analysis (GRA) method, and employs Fuzzy-set Qualitative Comparative Analysis (fsQCA) to reveal the collaborative pathways of multiple factors. The 2022 Government Affairs Index Weibo Influence Report issued by the Chinese government is used as the primary data source, selecting five dimensions: platform support, public participation, service level, response capacity, and social influence. Grey Relational Analysis (GRA) is then applied to verify the correlation between these five dimensions and the tripartite collaboration efficiency in government-platform-public interaction. The study finds that: (1) A positive social influence is a necessary condition for achieving high efficiency tripartite collaboration. A lack of interaction with netizens will result in low efficiency in government management of online public opinion. (2) If the government maintains proactive response capabilities, engages in high interaction with stakeholders such as netizens and the media, and improves the service level of both software and hardware in cyberspace, this represents the optimal combination pathway for enhancing tripartite collaboration efficiency. (3) If the government fails to focus on public satisfaction and social influence, even if local governments increase investments in software and hardware and improve service levels, effective management outcomes will not be achieved. This study’s innovation lies in the combined use of GRA and fsQCA to objectively identify the pathways for improving government-platform-public collaboration, providing scientific evidence for enhancing the efficiency of collaborative governance in online public opinion.

## Introduction

China’s rapid digital transformation, which is underpinned by over one billion Internet users and landmark innovations in e-government and social credit systems, has created a uniquely dynamic context for network governance. The interplay between centralized policy frameworks and burgeoning citizen-driven discourse on platforms like Weibo has both expanded channels for public participation and introduced novel regulatory challenges [1]. The internet constitutes a virtual societal ecosystem characterized by shared connectivity, decentralized architecture, and distributed interactions among heterogeneous digital actors [2]. Within this cyberspace, individuals, organizations, and institutional stakeholders collectively shape public opinion trajectories through complex interest networks, exerting transformative influences on economies, political authority, and cultural paradigms [3]. The evolution of online public opinion manifests as a dynamic propagation process where temporally sequenced interactions between stakeholders generate cascading effects [4]. Understanding these temporal spatial diffusion patterns—particularly the equilibrium mechanisms governing actor interdependencies on social media platforms—is critical for developing effective cyber governance frameworks [5].

Traditional network governance is defined as the process by which multiple autonomous actors within a loosely coupled network collaborate to manage public affairs through shared rules, protocols, and trust mechanisms [6]. The rise of converged media ecosystems, which integrate emerging and traditional media through the symbiosis of mobile internet, has intensified governance challenges [7]. These platforms facilitate real-time, omnidirectional information flows that disrupt conventional top-down regulatory approaches. Social media controversies frequently center on public interest issues ranging from environmental crises to healthcare accessibility, where virality directly correlates with citizen trust in institutional legitimacy, a determinant of social cohesion under public value theory [8,9].

This decentralized digital landscape necessitates reimagined governance models that reconcile bureaucratic efficiency with democratic participation. While public value theory acknowledges inherent tensions between administrative systems and pluralistic demands, the polycentric character of online discourse presents opportunities for collaborative value creation [10]. Traditional reactive governance mechanisms prove inadequate in steering opinion ecosystems shaped by networked value orientations and interest articulations [11]. A cooperative governance paradigm, aligning governmental interventions with stakeholder priorities through consensus-driven public value identification, emerges as an actionable framework for enhancing regulatory efficacy in this complex socio-technical environment [12]. In the digital era, network governance is reconceptualized as a dynamic system in which government, platforms, and citizens not only engage in collaborative decision making via real-time data exchange and feedback loops but must also balance algorithmic transparency, platform accountability, and public deliberation to contend with the exponential acceleration and reach of information flows [13].

In the process of network management, the government, platforms, and the public serve as the primary management entities, each bearing significant roles and responsibilities [14]. As the executor of public authority, the government has the duty to formulate policies and regulations, maintain social order, and safeguard public interests. Network platforms, as providers and operators of online services, hold vast amounts of user data and technical resources, giving them unique advantages and influence [15]. The public, as users and participants in cyberspace, directly influences the effectiveness of network governance through their behaviors and opinions [16].

Traditional public opinion governance in network management often relies on centralized authority (such as government-led monitoring and containment), but grassroots governments, due to their limited technical capacity, often adopt simple methods such as “blocking and deleting”, which conflict with the public’s expectations for transparency and participatory governance [17]. Therefore, effective network governance must embed robust accountability, transparency, and algorithmic-ethics safeguards to protect individual rights online [18]. Ethical algorithmic design further mandates fairness and non-discrimination, reinforcing institutional legitimacy and public trust [19].

To effectively address the challenges of managing cyberspace and promote its healthy development, it is urgent to reconstruct the roles of the government, platforms, and the public in network governance. This involves building a more efficient, rational, and orderly network governance system [20]. By clarifying the responsibilities and powers of each governance entity and strengthening communication and collaboration between them, a collaborative governance model involving multiple parties has become an important research topic and practical direction in the field of network management [21].

Effective cyber governance emerges from the interdependent contributions of governmental, platform, and public actors, each operating through distinct institutional mechanisms [22]. Governments enable collaboration through adaptive regulatory frameworks combining policy incentives, technological supervision systems, and interagency coordination protocols [23]. Platforms mediate governance efficacy via algorithmic content moderation and opinion stewardship, balancing commercial objectives with sociotechnical responsibilities. Under the state’s regulatory leadership and with platforms providing technical support, digitally literate citizens, who leverage accessible participatory channels, act as essential co-governors. Their timely contributions refine governance processes and strengthen both the legitimacy and adaptability of cyber governance frameworks [24]. The inherent tension between these stakeholders’ divergent interests—state prioritization of social stability, platform focus on value creation, and civic demands for rights protection—constitutes the central challenge in collaborative governance [25].

Scholars have conducted extensive and in-depth studies on the roles and functions of the government, platforms, and the public in network governance from various disciplinary perspectives and research methods. Regarding the role of the government in network management, some scholars emphasize the government’s leading position and regulatory responsibilities [26]. By formulating comprehensive laws, regulations, and policies, the government can regulate online behavior and maintain order and security in cyberspace. Government departments can also enhance public understanding of emergency situations through information dissemination, improving responses to online public opinion [27]. Tan (2020) [28] pointed out that the Singapore government chose social media as a tool for shaping public opinion, promoting public policies, and managing the internet. The government can use big data to improve its governance capabilities and compensate for the limitations of traditional methods of online public opinion management.

In contrast to the above views, Wang(2023) [18] argued that the supervision mechanism of the internet should be multi-agent and parallel, with the government, the internet, and individuals all sharing responsibility. In network management, the government should avoid excessive interference, focusing on collaboration and coordination with other management entities, and fully utilizing market mechanisms and social forces. For example, Savas(2022) [29]conducted a study from the perspective of governance tools and proposed that government public opinion governance in cyberspace should shift from coercion to guidance.

In guiding online public opinion, platforms play a key role by enabling governments to engage directly with the public via social media, whose openness facilitates the rapid achievement of governance goals [30]. Omoush(2023) [31] found that in the government’s use of social media, transparency, participation, and collaboration can enhance citizens’ trust in the government. Casero (2022) [32] systematically explored the role of platforms in governance and other public fields, discovering that platforms promote governance by connecting, expanding, mediating, and mobilizing. Kumar(2022) [33] believed that scientifically utilizing online platforms for communication can achieve a two-way flow of information, facilitating a harmonious society.

Scholars generally agree that the public is a crucial force in network management, with public participation and supervision playing an essential role in identifying and solving online issues [34]. Wang(2025) [35] argued that the public is not only the object of governance but also an active subject of governance, and thus, more voice and participation rights should be granted to the public. Moreover, the stronger citizens’ comprehension and mastery of digital technologies, the better the effectiveness of government governance, and such digital literacy can be enhanced through education [36]. Good governance theory further posits that a reduced distance between the public and government accelerates the public’s trust in government affairs media, thereby enhancing governance effectiveness [37]. Furthermore, informal civil society entities strengthen networked governance by co-producing digital public services and facilitating direct feedback loops. This role is further elaborated by Minbaeva(2023) [38], who notes that civil society organizations and NGOs reinforce such informal governance practices—specifically, by developing industry standards and participating in content review processes.

Although existing studies have made progress in understanding the roles of the government, platforms, and the public in network governance, there are still some deficiencies. On one hand, most research focuses on the study of individual actors, with relatively little attention paid to the collaborative governance mechanism among the government, platforms, and the public, as well as a lack of systematic analysis of multifactor collaborative governance. On the other hand, research on the specific pathways and methods for reconstructing the roles of these actors in network governance is still insufficient, and there is a lack of actionable suggestions.

This study investigates the configurational dynamics underlying tripartite government-platform-public collaboration through an integrated analytical framework. Confronting the inherent complexity of interdependent governance factors, we synergize Grey relational analysis (GRA) and Fuzzy-set qualitative comparative analysis (fsQCA) to transcend conventional single-factor paradigms. GRA first identifies critical determinants, namely social influence, public participation, response capability, service level, and platform support, by quantifying their systemic associations with collaborative efficiency. Building on this, fsQCA then elucidates how combinations of these factors yield high governance efficiency, revealing pathways for optimizing multi-stakeholder synergies. This methodological design addresses a critical gap in collaborative governance literature by systematically mapping causal complexity, where neither isolated variables nor uniform solutions sufficiently explain effectiveness. Our findings provide policymakers with contextually adaptive models that balance regulatory imperatives, technological mediation, and civic engagement logics.

## Research methodology and content

### Method

#### Grey relational analysis.

Grey relational analysis (GRA) quantifies the correlation degree between reference and comparative sequences through relational measurement, effectively reflecting inter-indicator associations [39]. Widely applied in management science, sociology, and economics, this study employs GRA to construct an influential factor model for tripartite collaborative governance efficiency from both external and internal dimensions. The implementation procedure comprises four steps.

Sequence Definition: The reference sequence represents Sequence Definition: The reference sequence represents tripartite collaboration efficiency, while comparative sequences encompass external factors (platform support, public participation, social influence) and internal factors (service level, response capacity). Data Normalization: To eliminate dimensional discrepancies, data standardization was performed using the mean value method, selected from three normalization approaches (unitary, initial value, and mean value methods). Grey Relational Coefficient Calculation: The relational coefficients between reference and comparative sequences were computed using the established GRA algorithm. Relational Degree Classification: Following prevalent academic classification standards, the relational degrees were categorized into four levels (Table 1) to facilitate systematic interpretation, while comparative sequences encompass external factors (platform support, public participation, social influence) and internal factors (service level, response capacity). Data Normalization: To eliminate dimensional discrepancies, data standardization was performed using the mean value method, selected from three normalization approaches (unitary, initial value, and mean value methods). Grey Relational Coefficient Calculation: The relational coefficients between reference and comparative sequences were computed using the established GRA algorithm. Relational Degree Classification: Following prevalent academic classification standards, the relational degrees were categorized into four levels (Table 1) to facilitate systematic interpretation.

**Table 1:** Classification criteria for GRA.

Grey relational degree	Classification criteria	Grey relational degree	Classification criteria
0.00-0.50	Weak Association	0.81-0.90	Strong Association
0.51-0.80	Moderate Association	0.91-1.00	Extreme Association

#### Fuzzy-set qualitative comparative analysis.

This study adopts Fuzzy-set qualitative comparative analysis(fsQCA) to explore sustainable development pathways for tripartite collaboration efficiency through a holistic perspective. Given the complexity of influencing factors and the small sample size (31 provincial-level regions in China) that aligns with QCA’s methodological requirements, fsQCA was selected to mitigate potential multicollinearity issues while examining conditional configurations. The analysis identifies core conditions and enhancement paths for high comprehensive intervention capability through comparative configuration analysis [40]. The six-step procedure includes.

Model Construction: Theoretical and empirical foundations guided the selection of conditional variables. Case Selection: The province’s meeting research criteria were systematically screened for analysis. Condition Outcome Calibration: Raw data were transformed into fuzzy-set membership scores using fsQCA 3.0 software. Necessity Analysis: Essential prerequisites for outcomes were identified through necessity testing. Configuration Analysis: Truth table refinement and standard analysis yielded solution terms and path combinations. Robustness Testing: Multiple sensitivity analyses ensured result reliability.

To leverage the complementary strengths of GRA and fsQCA, this study first applies GRA to quantify the degree of association between five antecedent conditions and the outcome variable. By ranking these relational degrees, GRA objectively identifies the most influential factors. These ranked values then inform the calibration of fuzzy-set membership scores in fsQCA, improving the consistency and empirical grounding of set thresholds and reducing subjective bias during calibration. The combined approach not only preserves GRA’s sensitivity to inter-indicator relationships but also exploits fsQCA’s capacity to reveal complex causal configurations. Consequently, this integration offers a robust mixed-methods framework for uncovering the multiple pathways that sustain high tripartite collaboration efficiency.

### Data sources

This study uses the survey data from the “2022 Government Affairs Weibo Influence Report” published by the People’s Daily Online Public Opinion Data Center, which covers 31 provinces in China. From this, five condition variables and one outcome variable are selected. The “2022 Government Affairs Weibo Influence Report”, provided by the People’s Daily Online Public Opinion Data Center, evaluates all officially certified Weibo accounts of institutions across China. The evaluation system includes: communicative power, service ability, interactivity, and recognition.

### Variable

#### Condition variables.

This paper selects five conditional variables, namely platform support, public participation, service level, response capacity, and social influence.

Platform support reflects the breadth of audience reach achieved by government Weibo accounts. A higher score denotes that government content is seen by a larger slice of netizens, underscoring the platform’s capacity to broadcast information widely. It is based on the “communicative power” metric from the 2022 Government Affairs Weibo Influence Report and captures the total number of post impressions, the count of active followers, and the active follower ratio.

Public participation measures the depth of citizen engagement with government Weibo content. This indicator distinguishes active user responses, such as discussions and endorsements, from mere viewership, highlighting meaningful two-way interaction. Derived from the report’s “Interactivity” metric, it aggregates the volume of shares, comments, and likes generated by verified or trusted accounts, alongside the engagement rate.

Service level gauges the extent to which government Weibo accounts deliver public services online. A higher Service level indicates that the government is leveraging social media as a direct service channel rather than solely as an information outlet. Based on the “service ability” metric, it comprises the count of proactive inquiry responses issued by official accounts, the total number of public-oriented posts, and the proportion of original content.

Response capacity evaluates how swiftly and effectively government Weibo accounts address citizen inquiries and issues. Faster, more comprehensive responses translate into a higher index, signaling stronger new-media governance capability. It corresponds to the “competitiveness index” in the report, calculated as the average competitiveness score across all prefectural regions within a province, focusing specifically on reply latency and follow-up actions.

Social influence captures netizen approval of government messaging on Weibo. A higher social recognition score indicates that citizens not only receive information but also identify with and trust the content’s legitimacy. Taken from the report’s “recognition” metric, it reflects the average endorsement score (positive sentiment ratings) of the top ten government institutional accounts in each province.

#### Outcome variable.

**Fig 1:**
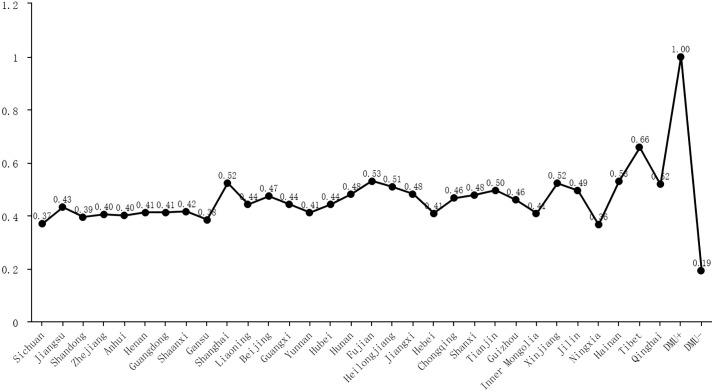
Results of tripartite collaboration efficiency values.

This study employs the input-oriented Data Envelopment Analysis (DEA) method under the constant-returns-to-scale assumption to derive a tripartite collaboration efficiency score for each province [41], with the calculation results presented in Fig 1. This method was selected because DEA accommodates multiple heterogeneous inputs and outputs without requiring a predetermined functional form. The DEA model uses three inputs: platform support, service level, and response capacity, because they reflect the resources and capacities contributed by the platform, government, and public to the collaboration. Public participation and social influence serve as the model’s outputs because effective collaboration among government, platforms and citizens should manifest in both active user engagement and broad social endorsement of governance initiatives.

By comparing each province to a best-practice frontier, the model transparently evaluates how effectively government, platform, and public actors jointly convert their inputs into collaborative outcomes. To ensure dimensional consistency with other variables, the calculated comprehensive efficiency values are scaled up by a constant multiplier, as this proportional adjustment preserves relative differences and does not compromise subsequent analyses.

## Analysis of the empirical results

### Descriptive statistics

This paper calculates the minimum, maximum, mean, and standard deviation for the sample data of the 31 provinces across the country, as shown in Table 2. These descriptive statistics provide the basis for setting calibration anchor points in subsequent analyses.

**Table 2:** Descriptive statistics of variables.

Variable	Code	Minimum	Maximum	Mean	Standard deviation
Platform support	PS	10.910	97.120	56.497	18.868
Service level	SL	33.520	91.150	66.205	12.615
Public participation	PP	9.570	93.210	62.017	19.281
Response capability	RC	20.810	92.930	61.827	16.001
Social influence	SI	38.011	73.560	64.567	9.115
Tripartite collaboration efficiency	TCE	62.040	179.576	139.520	28.001

**Fig 2:**
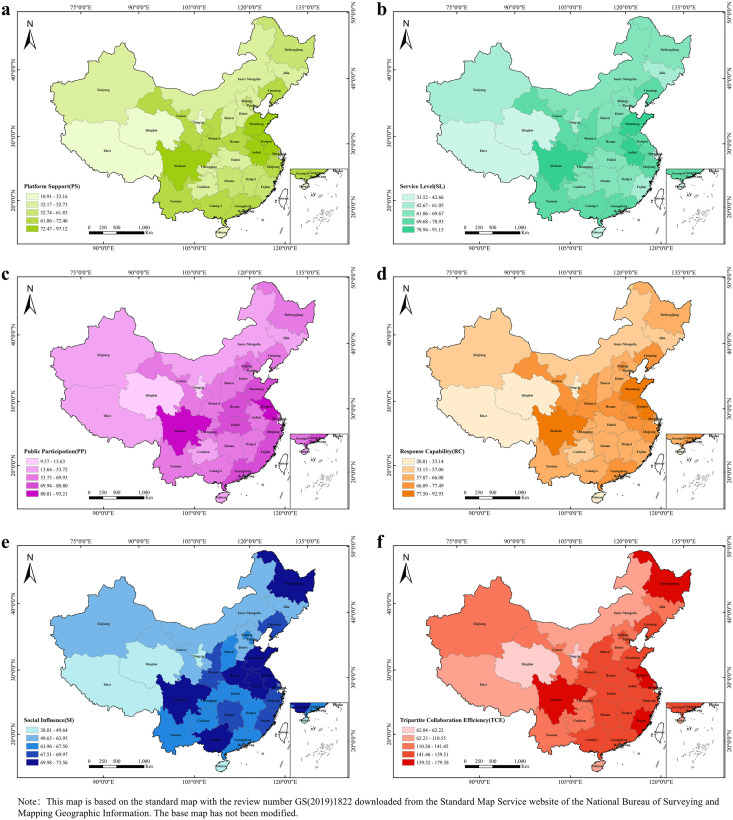
Comparison of condition variables and outcome variable values for each province.

An analysis of the five condition variables reveals substantial variation among provinces, as shown in Fig 2. A cross-sectional comparison of the condition variables reveals that the average platform support for local governments is 56.497, while the average values for service level, public participation, and social influence are all above 60, by upgrading technical infrastructure to enhance information dissemination reach and optimizing interface design to improve user accessibility.

Sichuan and Shanghai lead in platform support and service level, with scores of 97.12 and 91.15 for Sichuan and 67.60 and 60.65 for Shanghai. By contrast, Tibet records only 10.91 for platform support and 33.52 for service level. The gap between Sichuan and Tibet in platform support exceeds eighty-six points, while the difference in service level is more than fifty‑seven points. These disparities reflect differences in economic capacity and infrastructure. Sichuan’s urban broadband coverage is above ninety percent, enabling continuous system upgrades and dedicated technical teams. In Tibet, challenging geography and more limited fiscal resources restrict both network reach and the establishment of digital governance offices.

Public participation and social influence also vary widely. Jiangsu’s public participation score of 93.21 is more than ten times Qinghai’s 9.57. This contrast reflects Jiangsu’s high university enrollment rates and well-developed community Internet centers that foster digital literacy and civic engagement. In terms of social influence, Sichuan’s score of 92.93 is more than three times higher than Tibet’s 27.44. Sichuan’s diversified culture generates content that resonates across social media platforms, while lower urbanization and sparser mobile network coverage in Qinghai and Tibet limit online engagement and message amplification. Response capacity follows a similar pattern. Provinces such as Sichuan and Anhui exceed seventy in response capacity by deploying rapid-response units and automated monitoring. In Qinghai, the score falls to 38.01 because fewer IT staff lead to delays in addressing emerging issues.

The standard deviation for comprehensive efficiency is 28.001, with Shanghai showing the highest efficiency at 179.58, while Qinghai showing the lowest at 62.04, reflecting the imbalance in government-platform-public collaboration across China. Shanghai’s leading collaboration efficiency can be attributed to its strong economic foundation and comprehensive broadband coverage that facilitates seamless coordination across government departments. Jiangsu and Fujian achieve only marginally lower efficiency by coupling solid economic conditions with digital education and public engagement. These results indicate that enhancing collaboration efficiency requires more than investment in digital infrastructure. It also calls for systematic efforts to raise public digital skills and to strengthen institutional response mechanisms in provinces with less developed economies.

### Data calibration

The data were extracted from official statistical reports demonstrating high reliability. Given the absence of external theoretical calibration benchmarks, this study employs the direct calibration method, which establishes three theoretically grounded qualitative anchor points: fully affiliated thresholds (upper bounds), crossover points (intermediate values), and fully unaffiliated thresholds (lower bounds). Following established practices for skewed data distributions, calibration anchors were determined through percentile analysis: 95th percentile as fully affiliated thresholds, 50th percentile as intersection points, and 5th percentile as fully unaffiliated thresholds.

As shown in Table 3, these anchors were systematically applied to five conditional variables and one outcome variable. The calibration process transformed raw statistical data into fuzzy-set membership scores ranging from 0 to 1, where 1 indicates full set membership and 0 denotes complete non-membership. Cases with exact 0.5 membership scores received a 0.001 adjustment to prevent analytical ambiguities during logical minimization procedures.

**Table 3:** Calibration anchors for variables.

Category	Variable	Fully affiliated points	Intersection point	Fully unaffiliated points
Outcome	TCE	175	147	62
Conditions	PS	87	58	12
	SL	86	66	37
	PP	92	67	12
	RC	86	64	25
	SI	73	67	38

### Grey relational analysis

To further identify the influencing factors for tripartite collaboration, this paper employs Grey Relational Analysis (GRA). Grey Relational Analysis measures the degree of correlation between data, specifically the degree of association between the reference sequence and the comparison sequence, thus reflecting the relationships between indicators. In this study, the efficiency of government, platform, and public collaboration is used as the reference sequence, while external factors (platform support, public participation, and social influence) and internal factors (service level and response capability) serve as the comparison sequences. The grey relational coefficients between the reference sequence and the comparison sequences are calculated, with the results presented in Table 4.

**Table 4:** Correlation between government, platform, and public collaboration and related influencing factors.

Indicator	Correlation degree	Correlation level	Ranking
Social influence	0.848	Highly correlated	1
Public participation	0.732	Moderately correlated	2
Response capability	0.721	Moderately correlated	3
Service level	0.697	Moderately correlated	4
Platform support	0.657	Moderately correlated	5

As shown in Table 4, the correlation degree between social influence and the efficiency of tripartite collaboration is 0.848, which falls into the “Highly Correlated” category. This indicates that social influence caused by online public opinion events is a core indicator when examining the collaboration between the government, the platform, and the public. When online public opinion events generate positive social influence, they not only enhance the legitimacy of government actions but also encourage platforms to allocate more resources to content dissemination and public engagement, creating a virtuous cycle of collaboration. The correlation degrees between public participation, response capability, service level, platform support, and tripartite collaboration efficiency are 0.732, 0.721, 0.697, and 0.657, respectively, all of which are categorized as “Moderately Correlated”. This suggests that active citizen engagement and timely government reactions to public concerns are more critical drivers of collaborative efficiency than the mere provision of online services or platform infrastructure.

### Necessary conditions analysis

This paper uses the fsQCA 3.0 software to conduct necessary condition analysis on the calibrated data, examining whether single conditions are necessary for high or low efficiency in government-platform-public tripartite collaboration. The results are shown in Table 5. Consistency is an important indicator for determining necessary conditions, representing the degree of consistency between the sample test and the set relationship. When the consistency level is greater than 0.9, the condition is considered a necessary condition for the result. Coverage, on the other hand, represents the ratio of specific cases to the total number of cases and is used to assess the importance of antecedent condition combinations and the explanatory power of condition variables for the outcome variable.

**Table 5:** Necessary condition analysis for single variables.

Antecedent condition	High tripartite collaboration efficiency		Low tripartite collaboration efficiency	
	Consistency	Coverage	Consistency	Coverage
Platform support	0.848064	0.839817	0.642110	0.581590
~Platform support	0.577481	0.770528	0.823148	0.832082
Service level	0.806745	0.915885	0.688095	0.601109
~Service level	0.582361	0.852894	0.737322	0.777264
Public participation	0.880304	0.864080	0.619826	0.589834
~Public participation	0.605769	0.638225	0.911608	0.874466
Response capability	0.851893	0.671199	0.640084	0.586137
~Response capability	0.586622	0.635316	0.839354	0.838279
Social influence	0.941943	0.640545	0.654940	0.549519
~Social influence	0.508925	0.617229	0.838004	0.929588

Note: In qualitative comparative analysis, “~” represents the negation of the set operation, i.e., nonexistence.

From the calculations, it can be seen that for high tripartite collaboration efficiency, social influence has a strong explanatory power and is a necessary condition for evaluating the effectiveness of tripartite collaboration. For low efficiency, response capability as a single antecedent condition is a necessary condition. Consistency levels for the other factors are below 0.9 and need to be further explored through configuration analysis.

### Analysis of condition configurations

In this study, configuration analysis was performed using the fsQCA3.0 software. Based on the suggestions of scholars [42], the consistency threshold was set to 0.8, the case threshold to 1, and the natural break points were used as the standard for setting PRI (proportional reduction in inconsistency) thresholds. The PRI for the high-efficiency collaboration configurations was set at 0.75, and the PRI for the low-efficiency collaboration configurations at 0.5 (typically, a threshold no lower than 0.5 is sufficient).

A truth table analysis was conducted, followed by Boolean algebra operations for intermediate and reduced solutions to identify core and peripheral conditions. The configuration results are shown in Table 6. To ensure the interpretability of the conclusions, only configurations with coverage greater than 0.1 were analyzed to explore the important causal mechanisms of tripartite collaboration.

**Table 6:** Configurations for high and low tripartite collaboration efficiency.

Antecedent condition	High tripartite collaboration efficiency					Low tripartite collaboration efficiency	
	H1	H2	H3	H4	H5	L1	L2
Platform support	O	O	X	X	X	X	O
Service level		O	O	X	X		O
Public participation	•		•	•		**⊗**	**⊗**
Response capability	O	O	O	X	X	X	O
Social influence	•	•			•	**⊗**	**⊗**
Consistency	0.957	0.912	0.976	0.995	0.965	0.951	0.940
Raw coverage	0.802	0.786	0.510	0.497	0.17	0.735	0.452
Unique coverage	0.025	0.018	0.003	0.003	0.019	0.452	0.049
Overall coverage	0.880					0.793	
Overall consistency	0.901					0.954	

Note: “•“ indicates that the core condition exists, “**⊗**“ indicates that the core condition does not exist, “O” indicates that the edge condition exists, “X” indicates that the edge condition does not exist, and blank indicates that the condition may or may not exist.

Currently, five robustness check methods are commonly used in the academic community: adjusting the calibration values, raising the consistency threshold, adjusting the PRI threshold, adjusting case frequency, and adding other antecedent conditions. In general, one of these five robustness check methods can be chosen. Due to the small sample size and the lack of a clear basis for adjusting calibration anchor points, this study chose to enhance the case consistency threshold for robustness testing. The case consistency threshold was increased from 0.8 to 0.9, and the new configurations were consistent with the previous analysis results, indicating that the configuration results remained unchanged. This demonstrates that the data analysis results are robust.

From the calculations shown in Table 6, we can conclude that there are five configurations with high comprehensive intervention capability, namely H1, H2, H3, H4, and H5. This indicates that there are multiple paths to enhance tripartite collaboration efficiency. Among these, H2 and H5 share the same core conditions, as do H3 and H4, forming second-order equivalent configurations. The consistency for these five configurations is 0.995, 0.965, 0.976, 0.912, and 0.957, all of which exceed the consistency threshold of 0.8. The overall consistency is 0.901, which indicates that the analysis results are reliable. The provinces covered by these configurations include Sichuan, Jiangsu, Shandong, Zhejiang, Henan, Shanghai, Shaanxi, Guangdong, Beijing, Liaoning, Hunan, Fujian, Anhui, Guangxi, Hubei, Heilongjiang, Jiangxi, and Shanxi.

On the other hand, there are two configurations with low collaboration efficiency, namely L1 and L2. Both share the same core conditions, forming second-order equivalent configurations. The consistency of these solutions is 0.954, and the overall reliability is high. The provinces covered by these configurations include Qinghai, Hainan, Tibet, Ningxia, Jilin, Inner Mongolia, Xinjiang, Chongqing, Guizhou, Hebei, Tianjin, Gansu, and Yunnan.

### Comprehensive analysis between configurations

From the analysis above, the pathways for high and low tripartite collaboration efficiency are summarized as follows.

#### Pathways for high tripartite collaboration efficiency.

H1. Combination. Core conditions: public participation + social influence. Secondary conditions: platform support + response capacity. Implication: The government places particular emphasis on the level of interaction with netizens and social satisfaction. The higher the public participation, the more positive the social impact of the government’s intervention on public opinion issues. At the same time, the higher the platform support and the government’s ability to respond to public opinion, the more beneficial it is for improving government network management capabilities.

H2. Combination. Core condition: social influence. Secondary conditions: platform support + service level + response capacity. Implication: The government focuses on social impact and public satisfaction. Increasing platform support, improving service levels, and enhancing response capabilities are also effective ways to improve the government’s network management capacity. Moreover, strong response capacity ensures that public input is addressed promptly, creating a feedback loop that motivates sustained participation and strengthens trust in the collaborative system.

H3. Combination. Core condition: public participation. Secondary conditions: service level + response capacity. Implication: The government places special emphasis on interaction with netizens and has good public participation. Public participation is reinforced by high service levels—such as streamlined online service processes or tailored public services—that make citizens feel their engagement yields practical benefits, thereby increasing their willingness to participate actively. At the same time, improving service levels and response capabilities is an effective way to enhance the government’s network management capacity.

H4. Combination. Core condition: public participation. Implication: The government values public participation and positive interaction with the network. Even without additional supporting conditions, prioritizing public participation works because it transforms citizens from passive recipients of information into active co-creators of policies. Focusing on improving public participation is an effective way to enhance the government’s network management capacity.

H5. Combination. Core condition: social influence. Implication: The government places particular emphasis on the social impact of public opinion management. High social impact, driven by widespread public approval and positive sentiment, serves as a unifying force that bridges gaps between government, platforms, and citizens. Effective intervention to enhance social impact is an important way to improve the government’s network management capacity. This makes it an important way to improve the government’s network management capacity, as strong social impact can foster a shared commitment to collaboration.

#### Pathways for low tripartite collaboration efficiency.

L1. Combination. Noncore conditions: public participation + social influence. Implication: When public participation is low and the social impact of public opinion management is weak, this combination hinders the improvement of tripartite collaboration efficiency. The absence of public participation and effective social impact will result in low government network management capability.

L2. Combination. Noncore conditions: public participation + social influence. Missing secondary conditions: platform support + service level + response capacity. Implication: If network managers do not value public participation or the social impact of public opinion management, and at the same time lack platform support, service levels, and response capacity, this combination will lead to low tripartite collaboration efficiency in network governance.

## Conclusions and recommendations

### Conclusions

This study identifies five pathways to high tripartite collaboration efficiency and two pathways leading to low efficiency, offering critical insights into the dynamics of government-platform-public collaboration in network governance. This study contributes to understanding the configurational nature of collaborative efficiency, highlighting that no single condition guarantees success or failure, but their strategic combination determines outcomes.

First, public participation emerges as a pivotal driver of high efficiency, featured as a core condition in three out of five high-efficiency pathways. Its effectiveness is amplified when paired with strong social influence (H1), service level, and response capacity (H3), or even independently (H4). This underscores that active citizen engagement—whether through feedback, discussions, or co-creation—serves as a foundational pillar for enhancing collaborative outcomes, as it aligns government actions with public needs and fosters trust in the collaborative system.

Second, social influence plays a dual role: it functions as a core condition alongside public participation in H1 and as the sole core condition in H5, highlighting its ability to unify stakeholders and bridge gaps between government, platforms, and the public. High social impact, driven by positive public sentiment and widespread approval, compensates for potential deficiencies in other conditions, reinforcing that legitimacy and public recognition are critical for sustaining collaboration.

In contrast, the low-efficiency pathways (L1-L2) reveal that insufficient public participation and weak social impact are key barriers to effective collaboration. L1 demonstrates that even in the absence of other deficiencies, low public engagement and negligible social influence directly hinder efficiency. L2 further emphasizes that combining these weaknesses with inadequate platform support, service level, and response capacity exacerbates inefficiencies, indicating that neglecting both core drivers and supporting conditions creates a “vicious cycle” in network governance.

### Recommendations

Building on the findings that tripartite collaboration efficiency hinges on public participation, social influence, and supportive conditions, targeted actions by governments, platforms, and the public are critical to translating these insights into practice.

For governments, prioritizing public participation requires establishing accessible, multi-channel engagement mechanisms—such as interactive portals, community forums, and mobile apps—that welcome diverse voices, and ensure their input directly informs policy. To amplify social impact, governments should develop strategic communication frameworks that highlight collaborative successes and use real-time sentiment analysis to refine messaging, bolstering public trust.

Platforms play a pivotal role in facilitating collaboration by enhancing accessibility, which includes designing user-friendly interfaces for diverse groups, integrating with mainstream social media to expand reach, and ensuring stability during peak engagement. They should also act as data intermediaries, providing governments with anonymized metrics on user behavior and topic trends to guide decision-making, while using algorithms to surface high-priority public concerns.

The public’s active engagement is foundational to effective collaboration. Citizens should leverage official platforms to report issues, join discussions, and spread verified information—reducing reliance on unregulated channels. Encouraging participation from underrepresented groups ensures inclusivity, paired with demands for transparent data sharing, fosters mutual responsibility and drives continuous improvement in the collaborative process.

In conclusion, reconstructing the tripartite collaboration between government, platform, and the public in network management is an essential requirement for improving management effectiveness and ensuring the healthy and orderly development of cyberspace. By establishing scientific and reasonable collaboration relationships, improving communication and coordination mechanisms, optimizing interest balance mechanisms, strengthening accountability mechanisms, and enhancing public participation capabilities, the three parties can collaborate effectively, creating a new pattern of construction, co-governance, and shared benefits in network management.

### Limitation

This study demonstrates that the integrated GRA and fsQCA framework offers a flexible tool for unpacking the configuration of government, platform, and public capabilities that underpin effective network governance. By adjusting grey relational reference values to reflect the correlation between variables and redefining fuzzy-set calibration thresholds, this approach can reveal how alternative stakeholder combinations shape collaborative efficiency across different institutional landscapes. Furthermore, ensuring algorithmic transparency, protecting user privacy, and upholding digital rights are fundamental to any governance model that aims to empower citizens as active co-governors rather than passive recipients.

Nonetheless, this study is limited by its exclusive focus on the Chinese network environment, which may constrain the generalizability of our findings to other national or democratic systems. Future research should extend this analysis to multiple platforms of varying governance structures. Comparative studies across different political regimes will be essential to uncover how institutional variations influence the pathways to efficient, legitimate, and inclusive network governance.

## Supporting information

S1 Data The data used in this paper for discussion and analysis. (CSV)

S2 File fsQCA3.0 software calculation result. (DOCX)
