# Supplementary material for: Pathways to effective network governance: A fuzzy-set QCA study of tripartite collaboration efficiency with Chinese official Weibo data
Source: PLoS One. 2025 Oct 7;20(10):e0331007. doi: 10.1371/journal.pone.0331007 (PMC12503339; doi:10.1371/journal.pone.0331007)
Supplement: S2 File — (DOCX) [file pone.0331007.s002.docx]

Appendix 1: Using fsQCA3.0 software calculation result output

Analysis of Necessary Conditions

Outcome variable: TCE

Conditions tested:

Consistency Coverage

PS 0.848064 0.839817

SL 0.806745 0.770528

PP 0.880304 0.915885

RC 0.851893 0.852894

SI 0.941943 0.864080

~PS 0.577481 0.638225

~SL 0.582361 0.671199

~PP 0.605769 0.635316

~RC 0.586622 0.640545

~SI 0.508925 0.617229

Analysis of Necessary Conditions

Outcome variable: ~TCE

Conditions tested:

Consistency Coverage

PS 0.642110 0.581590

SL 0.688095 0.601109

PP 0.619826 0.589834

RC 0.640084 0.586137

SI 0.654940 0.549519

~PS 0.823148 0.832082

~SL 0.737322 0.777264

~PP 0.911608 0.874466

~RC 0.839354 0.838279

~SI 0.838004 0.929588

**********************

*TRUTH TABLE ANALYSIS*

**********************

PRI0.75

File: C:/Users/wu/Desktop/5个变量的分析数据（处理后）.csv

Model: TCE = f（PS, SL, PP, RC, SI）

Algorithm: Quine-McCluskey

--- COMPLEX SOLUTION ---

frequency cutoff: 1

consistency cutoff: 0.954521

raw unique

coverage coverage consistency

---------- ---------- ----------

~PS*SL*PP*RC 0.510222 0.00562048 0.976362

PS*SL*RC*SI 0.786301 0.0184671 0.911963

PS*PP*RC*SI 0.801927 0.0340931 0.956605

~PS*~SL*PP*~RC*~SI 0.415045 0.00432342 0.994083

~PS*~SL*~PP*~RC*SI 0.48422 0.0191464 0.963145

solution coverage: 0.87913

solution consistency: 0.901285

Cases with greater than 0.5 membership in term ~PS*SL*PP*RC: 湖北 （0.53,0.47）,

黑龙江 （0.501,0.89）

Cases with greater than 0.5 membership in term PS*SL*RC*SI: 四川 （0.93,0.85）,

山东 （0.9,0.71）, 安徽 （0.82,0.55）, 江苏 （0.79,0.93）,

浙江 （0.78,0.65）, 河南 （0.68,0.61）, 陕西 （0.63,0.501）,

辽宁 （0.63,0.6）, 广西 （0.57,0.53）, 广东 （0.51,0.49）

Cases with greater than 0.5 membership in term PS*PP*RC*SI: 四川 （0.93,0.85）,

江苏 （0.86,0.93）, 山东 （0.82,0.71）, 浙江 （0.78,0.65）,

河南 （0.68,0.61）, 上海 （0.65,0.97）, 陕西 （0.59,0.501）,

广东 （0.51,0.49）, 北京 （0.51,0.79）, 辽宁 （0.501,0.6）,

湖南 （0.501,0.73）, 福建 （0.501,0.94）

Cases with greater than 0.5 membership in term ~PS*~SL*PP*~RC*~SI: 江西 （0.51,0.64）

Cases with greater than 0.5 membership in term ~PS*~SL*~PP*~RC*SI: 山西 （0.54,0.49）

**********************

*TRUTH TABLE ANALYSIS*

**********************

File: C:/Users/wu/Desktop/5个变量的分析数据（处理后）.csv

Model: TCE = f（PS, SL, PP, RC, SI）

Algorithm: Quine-McCluskey

--- PARSIMONIOUS SOLUTION ---

frequency cutoff: 1

consistency cutoff: 0.954521

raw unique

coverage coverage consistency

---------- ---------- ----------

PP 0.880304 0.0234699 0.915885

SI 0.941943 0.0851091 0.86408

solution coverage: 0.965413

solution consistency: 0.838124

Cases with greater than 0.5 membership in term PP: 江苏 （0.96,0.93）,

四川 （0.95,0.85）, 浙江 （0.84,0.65）, 山东 （0.82,0.71）,

上海 （0.78,0.97）, 河南 （0.77,0.61）, 北京 （0.75,0.79）,

广东 （0.69,0.49）, 湖北 （0.66,0.47）, 福建 （0.66,0.94）,

陕西 （0.59,0.501）, 黑龙江 （0.55,0.89）, 江西 （0.55,0.64）,

辽宁 （0.501,0.6）, 湖南 （0.501,0.73）

Cases with greater than 0.5 membership in term SI: 黑龙江 （0.96,0.89）,

四川 （0.93,0.85）, 安徽 （0.93,0.55）, 山东 （0.91,0.71）,

河南 （0.9,0.61）, 广西 （0.9,0.53）, 江苏 （0.87,0.93）,

辽宁 （0.82,0.6）, 福建 （0.8,0.94）, 浙江 （0.78,0.65）,

上海 （0.78,0.97）, 湖南 （0.76,0.73）, 陕西 （0.63,0.501）,

山西 （0.56,0.49）, 广东 （0.51,0.49）, 北京 （0.51,0.79）

**********************

*TRUTH TABLE ANALYSIS*

**********************

File: C:/Users/wu/Desktop/5个变量的分析数据（处理后）.csv

Model: TCE = f（PS, SL, PP, RC, SI）

Algorithm: Quine-McCluskey

--- INTERMEDIATE SOLUTION ---

frequency cutoff: 1

consistency cutoff: 0.954521

Assumptions:

SI （present）

raw unique

coverage coverage consistency

---------- ---------- ----------

~PS*~SL*PP*~RC 0.497128 0.00308818 0.995055

~PS*~SL*~RC*SI 0.516892 0.0185288 0.965394

~PS*SL*PP*RC 0.510222 0.0032118 0.976362

PS*SL*RC*SI 0.786301 0.0184672 0.911963

PS*PP*RC*SI 0.801927 0.0254464 0.956605

solution coverage: 0.879748

solution consistency: 0.901348

Cases with greater than 0.5 membership in term ~PS*~SL*PP*~RC: 江西 （0.52,0.64）

Cases with greater than 0.5 membership in term ~PS*~SL*~RC*SI: 山西 （0.54,0.49）

Cases with greater than 0.5 membership in term ~PS*SL*PP*RC: 湖北 （0.53,0.47）,

黑龙江 （0.501,0.89）

Cases with greater than 0.5 membership in term PS*SL*RC*SI: 四川 （0.93,0.85）,

山东 （0.9,0.71）, 安徽 （0.82,0.55）, 江苏 （0.79,0.93）,

浙江 （0.78,0.65）, 河南 （0.68,0.61）, 陕西 （0.63,0.501）,

辽宁 （0.63,0.6）, 广西 （0.57,0.53）, 广东 （0.51,0.49）

Cases with greater than 0.5 membership in term PS*PP*RC*SI: 四川 （0.93,0.85）,

江苏 （0.86,0.93）, 山东 （0.82,0.71）, 浙江 （0.78,0.65）,

河南 （0.68,0.61）, 上海 （0.65,0.97）, 陕西 （0.59,0.501）,

广东 （0.51,0.49）, 北京 （0.51,0.79）, 辽宁 （0.501,0.6）,

湖南 （0.501,0.73）, 福建 （0.501,0.94）

**********************

*TRUTH TABLE ANALYSIS*

**********************

PRI=0.5

File: C:/Users/wu/Desktop/5个变量的分析数据（处理后）.csv

Model: ~TCE = f（PS, SL, PP, RC, SI）

Algorithm: Quine-McCluskey

--- COMPLEX SOLUTION ---

frequency cutoff: 1

consistency cutoff: 0.939691

raw unique

coverage coverage consistency

---------- ---------- ----------

~PS*~PP*~RC*~SI 0.734688 0.33088 0.951049

PS*SL*~PP*RC*~SI 0.452428 0.0486192 0.939691

solution coverage: 0.783307

solution consistency: 0.953947

Cases with greater than 0.5 membership in term ~PS*~PP*~RC*~SI: 青海 （0.95,0.95）,

海南 （0.86,0.86）, 西藏 （0.85,0.83）, 宁夏 （0.84,0.95）,

吉林 （0.65,0.67）, 内蒙古 （0.62,0.78）, 新疆 （0.58,0.6）,

重庆 （0.55,0.55）, 贵州 （0.53,0.61）, 河北 （0.52,0.67）,

天津 （0.52,0.44）

Cases with greater than 0.5 membership in term PS*SL*~PP*RC*~SI: 甘肃 （0.53,0.63）,

云南 （0.51,0.61）

**********************

*TRUTH TABLE ANALYSIS*

**********************

File: C:/Users/wu/Desktop/5个变量的分析数据（处理后）.csv

Model: ~TCE = f（PS, SL, PP, RC, SI）

Algorithm: Quine-McCluskey

--- PARSIMONIOUS SOLUTION ---

frequency cutoff: 1

consistency cutoff: 0.939691

raw unique

coverage coverage consistency

---------- ---------- ----------

~PP*~SI 0.796137 0.796137 0.954656

solution coverage: 0.796137

solution consistency: 0.954656

Cases with greater than 0.5 membership in term ~PP*~SI: 宁夏 （0.95,0.95）,

青海 （0.95,0.95）, 海南 （0.86,0.86）, 西藏 （0.85,0.83）,

内蒙古 （0.69,0.78）, 吉林 （0.65,0.67）, 新疆 （0.58,0.6）,

重庆 （0.55,0.55）, 甘肃 （0.53,0.63）, 河北 （0.53,0.67）,

贵州 （0.53,0.61）, 天津 （0.52,0.44）, 云南 （0.51,0.61）

**********************

*TRUTH TABLE ANALYSIS*

**********************

File: C:/Users/wu/Desktop/5个变量的分析数据（处理后）.csv

Model: ~TCE = f（PS, SL, PP, RC, SI）

Algorithm: Quine-McCluskey

--- INTERMEDIATE SOLUTION ---

frequency cutoff: 1

consistency cutoff: 0.939691

Assumptions:

~PP （absent）

raw unique

coverage coverage consistency

---------- ---------- ----------

~PS*~PP*~RC*~SI 0.734688 0.33088 0.951049

PS*SL*~PP*RC*~SI 0.452428 0.0486192 0.939691

solution coverage: 0.783307

solution consistency: 0.953947

Cases with greater than 0.5 membership in term ~PS*~PP*~RC*~SI: 青海 （0.95,0.95）,

海南 （0.86,0.86）, 西藏 （0.85,0.83）, 宁夏 （0.84,0.95）,

吉林 （0.65,0.67）, 内蒙古 （0.62,0.78）, 新疆 （0.58,0.6）,

重庆 （0.55,0.55）, 贵州 （0.53,0.61）, 河北 （0.52,0.67）,

天津 （0.52,0.44）

Cases with greater than 0.5 membership in term PS*SL*~PP*RC*~SI: 甘肃 （0.53,0.63）,

云南 （0.51,0.61）
